# Impact of Surface Properties of Core Material on the Stability of Hot Melt-Coated Multiparticulate Systems

**DOI:** 10.3390/pharmaceutics13030366

**Published:** 2021-03-10

**Authors:** Sonja Schertel, Sharareh Salar-Behzadi, Andreas Zimmer

**Affiliations:** 1 Department of Pharmaceutical Technology and Biopharmacy, Institute of Pharmaceutical Science, University of Graz, 8010 Graz, Austria; sonja.schertel@hermes-pharma.com (S.S.); sharareh.salar@rcpe.at (S.S.-B.); 2 Hermes Arzneimittel GmbH, Division Hermes Pharma, 82049 Pullach, Germany; 3 Research Center Pharmaceutical Engineering GmbH, 8010 Graz, Austria

**Keywords:** hot melt coating, lipid-based formulation, product instability, surface energy characteristics, crystal growth, phase separation

## Abstract

Hot melt coating (HMC) of an active pharmaceutical ingredient (API) powder with lipid-based excipients is an innovative method for manufacturing patient-convenient dosage forms. However, drug release instability is still its main industrial challenge. The correlation between the unstable pharmaceutical product performance with the solid-state alteration of lipids is currently well-investigated. The remaining problem is the inconsistent release alteration of different APIs coated with the same lipid after storage, such as faster release in some cases and slower release in others. The interaction between API surface and lipid-based coating and its alteration during storage were investigated in this work. The surface properties of five different APIs and the coating composition of tripalmitin and polysorbate 65 were screened via Washburn and pendant drop methods, respectively. Metformin hydrochloride and hydrochlorothiazide particles were each coated with the coating composition. The water sorption alteration of coated particles and the crystal growth of tripalmitin in the coating after storage were measured via tensiometry and X-ray diffraction. The cleavage work necessary to overcome the adhesion of coating composition on the core surface was calculated for each API. The accelerated release of the polar core (metformin) after storage was correlated with a low cleavage work and a distinctive phase separation. In contrast, a decelerated release of the hydrophobic core (hydrochlorothiazide) was favored by the crystal growth of the lipid-based coating. The gained knowledge can be used to design the product stability during the formulation development.

## 1. Introduction

Direct hot melt coating of API powder without pretreatment to granules or pellets is an innovative and economical method for manufacturing multiparticulate systems (MPS) as patient-convenient dosage forms. MPS are easy to swallow, and their manufacturing requires a smaller amount of excipients than other oral dosage forms, e.g., tablets. Moreover, the lipid-based coating provides suitable taste masking and a modified release [1,2]. The challenge of lipid-based formulations is, however, their instability during storage [3]. During the last decades, a large body of investigations showed the correlation between the monotropic polymorphism of lipids and the instability of their pharmaceutical products [3,4,5,6,7,8,9,10]. Beyond this, the relationship between alteration in the microstructure level, including crystal growth and phase separation, and the unstable product performance was investigated in our group [3,6]. In previous works, we developed a two-phasic lipid-based coating formulation composing tripalmitin (PPP) and polysorbate 65 (PS 65) for hot melt coating of N-acetylcysteine (NAC) crystals [1,3].

Taste-masked MPS with stable modified release could be achieved by tailoring the ratio of PPP and PS 65 as the coating. The induced polymorphic transformation of PPP from unstable α-form to the most stable β-form as the function of PS 65 ratio and temperature was shown immediately after manufacturing [1]. Further, despite stable polymorphic form, crystal growth of PPP and phase separation of the PPP/PS 65 coating system after storage under accelerated conditions occurred, causing faster NAC release after storage [3]. Recently, using the same coating composition of PPP/PS 65 for different APIs, we observed an inconsistent behavior in the release alteration after storage. There was a faster release in some cases and a slower release in others. This study investigated this phenomenon to provide useful knowledge of the interactions between different APIs as core materials with the lipid-based coating formulation. Two APIs with different polarities were used to provide hot melt-coated MPS. Alterations in the water sorption behavior and the API release after storage were investigated. Small-angle x-ray scattering provided information about the crystal growth of PPP.

Furthermore, the surface energy characteristics such as the surface energy and surface tension of five different APIs and the coating formulation PPP/PS 65 were investigated under stress factors, temperature, and relative humidity. Based on the surface energy characteristics, an approach was developed to predict the affinity between API cores with different lipophilicity and coating. This study generates a more profound knowledge and understanding of the instability mechanisms of coated MPS.

## 2. Materials and Methods

### 2.1. Materials

Hermes Arzneimittel GmbH (Pullach, Germany) kindly provided the APIs and the coating material. PharmaZell GmbH (Raubling, Germany) delivered N-acetylcysteine (NAC). Caffeine anhydrous granules were from Siegfried Pharma Chemikalien Minden GmbH (Minden, Germany) and acetylsalicylic acid (ASA) 40–80 mesh from Shandong Xinhua Pharmaceutical Co. LTD (Zibo, China). Hydrochlorothiazide (HCT) and metformin HCl (MET) were acquired from Suzhou Lixin Pharmaceutical Co., Ltd. (Suzhou, China) and Shouguang Fukang Pharmaceutical Co., Ltd. (Shouguang, China), respectively.

Magnesium stearate LIGAMED MF-2-V E470 was obtained from Peter Greven Nederland C.V. (Venlo, Nederland). Tripalmitin (PPP, melting point: 63–68 °C), commercially available as Dynasan^®^116, was purchased from IOI Oleo GmbH (Witten, Germany). Polysorbate 65 (PS 65, melting point: 33.3 °C, HLB = 10.5), available as Tween^TM^65, was obtained from Croda International Plc (Snaith, UK). Double distilled, deionized water was used (Ultrapure Water System, Milli-Q Merck Life Science, Darmstadt, Germany). The liquid α-bromonaphthalene (95% purity) was purchased from TCI Deutschland GmbH (Eschborn, Germany), and ethylene glycol (99% purity) and n-hexane (99% purity) were achieved from Carl Roth GmbH + Co. KG (Karlsruhe, Germany). The PTFE foil was obtained from Walfront (Wuhan, China).

**Figure 1:**
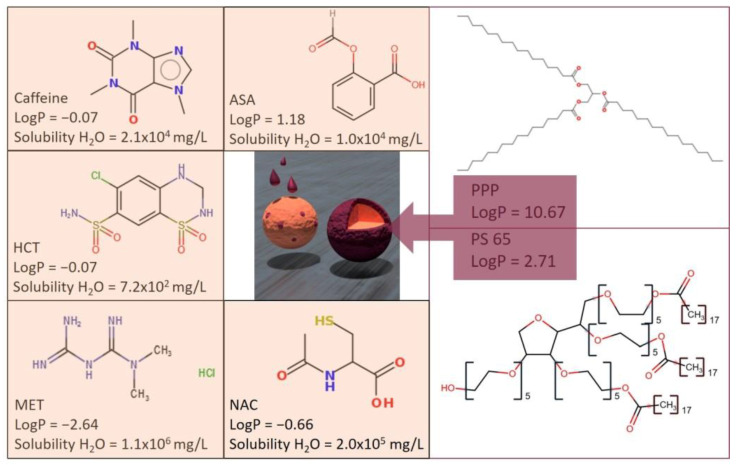
Chemical structures, logP-value, and water solubility of investigated APIs and coating material. Data were achieved from PubChem [11], and chemical structures were drawn by Molinspiration [12].

Figure 1 depicted the chemical structures and solubility data of selected APIs and coating materials.

### 2.2. Methods

This study investigated the affinity between the API core and the lipid-based coating and the surface energy characteristics for MET, HCT, caffeine, NAC, and ASA with different hydrophilic and lipophilic behaviors. Moreover, HCT and MET acted as core materials and were hot melt-coated with a mixture of PPP and PS 65. The coated MPS were investigated in terms of APIs’ release behavior from the coating and release alteration after storage, the water sorption behavior, and the crystal growth of PPP in the coating.

#### 2.2.1. Hot Melt Coating Process

The HMC process was carried out in the fluid bed coater Romaco Innojet laboratory system (Ventilus^®^ 2.5) equipped with a Romaco Innojet Hot-Melt-Device IHD-1 (Romaco Innojet GmbH, Steinen, Germany). The needle-shaped MET with a particle size of x_50_ = 239.04 ± 2.95 µm and the platelet-shaped HCT with an x_50_-value of 204.10 ± 6.00 µm were used as received, without any pretreatment to granules or pellets, as core material. The batch size was 225 g. MET with an angle of repose of 26.6–35.0° possessed a good flowability according to USP 43 (<1174> powder flow). The poor flowability of HCT with an angle of repose of 43.33 ± 1.24° was improved by adding 1% of magnesium stearate to the HCT powder to reach a fair flowability (angle of repose = 37.2°, fair, aid not needed).

MET batches were coated with a mixture of PPP and PS 65 in a ratio of 90 to 10 and 80 to 20, respectively, to gain 60%_*w/w*_ coating for each batch. The two batches of HCT were coated with the same ratios of PPP and PS 65 but to gain 25%_*w/w*_ of the coating. The coating amount was selected according to the hydrophilic behavior of the two APIs to guarantee a visible release alteration after storage.

The coating mixture was melted at 90 °C. The room condition for the HMC trials was 23 ± 1 °C and 50 ± 3% RH. The following process parameters were selected: inlet air temperature of 20 °C, atomizing air pressure of 0.9 bar, and a spray rate of 7 g/min. The airflow was varied between 30 (HCT) and 35 (MET) m^3^/h for each core material to provide the best fluidization in the process container.

The coated batches of MET and HCT were used to investigate the alteration in water sorption behavior and crystal growth of PPP and their correlation with the alteration in the API release after storage under long term (25 °C and 75% RH) and accelerated conditions (40 °C and 75% RH).

#### 2.2.2. In Vitro Release Profile of HCT and MET

A paddle apparatus II (Ph. Eur. 2.9.3) (Erweka DT826 LH, ERWEKA GmbH, Langen, Germany) at 37 ± 0.5 °C and 50 rpm was used. The dissolution tester was equipped with an automatic sampling station ASS8, a peristaltic pump IPC 8, and an FRL624 fraction collector. The dissolution media was 900 mL 0.1 N HCl. The samples of 1.5 mL were taken automatically and analyzed via Waters Acquity UPLC H-Class equipped with a UV/Vis detector (Waters GmbH, Eschborn, Germany). The column Waters Acquity UPLC BEH C18 1.7 µm, 2.1 × 150 mm was set to 40 °C. The elution of 0.3 µL (MET) and 3 µL (HCT) in a mobile phase gradient of 0.1% phosphoric acid and acetonitrile with a flow rate of 0.3 mL/min provided the calculation of released MET (absorbance at 221.5 nm) and HCT (absorbance at 271.0 nm). The UV absorption spectra of HCT and MET are shown in Figure S1 in the Supplementary Material.

#### 2.2.3. Small-Angle X-ray Scattering

The molecular arrangement and the number of stacked lamellae were investigated with a point-focusing camera system S3-MICRO from Bruker AXS (Karlsruhe, Germany), respectively. It contains two linear position detectors in the ranges of 10–1500 Å (SAXS) and 3.3–4.9 Å (WAXS) powered by the X-ray CuKα-radiation micro-source with a wavelength of 1.542 Å. The glass capillary with a diameter of approx. 2 mm filled with the coated MPS sample was placed into the capillary rotation unit. The exposure time of the filled capillary and a blank capillary measurement was 1300 s at room temperature. Samples were measured in triplicate.

The diffraction of coherent X-rays on groups of atomic planes, separated by a distance d, shows an interference pattern which can be explained by Bragg’s law,

(1) $$d=\;\frac{n\;\lambda }{2\;\sin\theta }$$

where λ is the X-ray wavelength (1.542 Å), θ is the diffraction angle, and n is the diffraction order [13,14].

The Scherrer equation calculated the average lamellae thickness of the molecular crystal structure D,

(2) $$D=\;\frac{K\lambda }{FWHM\;\cos\theta }$$

where λ is the wavelength, θ the diffraction angle, and K is the Scherrer constant, which depends on the crystal shape [14]. The K value of 0.9 is an appropriate approximation in the absence of detailed shape information [3]. The Full Width at Half Maximum (FWHM) of the SAXS peak (001 plane) is required for the Scherrer equation, which is calculated with a Gaussian amplitude function. The SAXS pattern of coated MPS HCT1, HCT2, MET1, MET2 at time zero (T0) and after the storage of one month and three months at 25 °C/60% RH and 40 °C/75% RH are depicted in Figure S2 in the Supplementary Material. The number of stacked lipid lamellae was calculated from the ratio of D and d. The Scherrer analysis is applied for nanoscaled particles smaller than 100 µm [14].

#### 2.2.4. Monitoring Phase Separation of the Coating Mixture via Confocal Raman Spectroscopy

HCT and MET powders were each pressed to an 18 mm tablet and coated with 100 µL of the molten mixture of PPP and PS 65 in equal ratio.

The Bruker Senterra confocal Raman spectroscope (Bruker Optik GmbH, Ettlingen, Germany) equipped with a motorized XYZ-Stage for chemical mapping, three magnifications (4×, 20×, and 50×), and a Lumenera Infinity 1B digital camera was used. A laser source of 785 nm with a power of 100 mW was beamed onto the sample through a pinhole aperture of 25 µm. The resolution of 3–5 cm^−1^ and an integration time of 30 s provided spectra recording. A measuring grid of 3 × 3 spectra was examined from each sample at a distance of 20 µm in the x and y-direction. In the z-direction, there was another measuring grid at 25 µm below the surface. Raman spectra were recorded after focusing on the sample with the 20× magnification at the wavenumber range of 1530 to 400 cm^−1^_._ The reference spectra of HCT, MET, PPP, and PS 65 were recorded under the same measuring conditions.

To remove the baseline drift of spectra, an asymmetric least squares smoothing baseline correction was performed for each spectrum with an asymmetric factor of 0.0001, a threshold of 0.0002, a smoothing factor of 8, and 5 iterations. The normalization step depended on the API system since it suppressed the API signal and highlighted PPP and PS 65. Therefore, spectra of HCT tablets and the appropriate reference powder spectra ($I_{\mathrm{PPP}}$ and $I_{\mathrm{PS}\;65}$) were normalized to their intensity value at 903 cm^−1^, where HCT has a firm peak. This peak can be assigned to the sulfonamide functional group [15].

A similar procedure was performed for the spectra of the MET tablets and the reference spectra PPP and PS 65. The normalization peak was at 738 cm^−1^, where MET has a strong peak due to the N-H wagging vibrations [16]. To extract the content of PPP and PS 65 in the MET and HCT tablet composition, respectively, a weighting function Equation (3) with the appropriate reference spectra ($I_{\mathrm{PPP}}$ and $I_{\mathrm{PS}\;65}$) was fitted to the measured spectrum of the coated tablets ($I_{\mathrm{PPP}+\mathrm{PS}\;65}$). The normalized Raman spectra ($I_{\mathrm{PPP}}$, $I_{\mathrm{PS}\;65}$, $I_{\mathrm{PPP}+\mathrm{PS}\;65}$) are depicted in the Supplementary Material (Figure S3). The distribution of the different components corresponds to the proportional weighing factors of PPP ($f_{\mathrm{PPP}}$) and PS 65 ($f_{\mathrm{PS}\;65}$).

(3) $$I_{\mathrm{PPP}+\mathrm{PS}\;65}=\;I_{\mathrm{PPP}}*\;f_{\mathrm{PPP}}+\;I_{\mathrm{PS}\;65}*\;f_{\mathrm{PS}\;65}$$

#### 2.2.5. Investigation of the Surface Energy of APIs and Their Water Sorption Behavior via Tensiometry

The Young and Dupré equation describes the contact between a liquid unit and a solid unit. It points out the relationship between the solid-vapor $\gamma_{\mathrm{sv}}$, liquid-vapor $\gamma_{\mathrm{lv}}$_,_ and solid-liquid $\gamma_{\mathrm{sl}}$ if the vapor (v) is in equilibrium with the solid (s) and the liquid [17,18,19,20,21].

(4) $$\gamma_{\mathrm{sv}}=\;\gamma_{\mathrm{sl}}+\;\gamma_{\mathrm{lv}}*\cos\theta$$

A contact angle θ between 0 and 90° shows wetting behavior. 0° is related to perfect wetting, whereas contact angles θ above 90° tend to avoid wetting [20].

The surface energy ($\gamma_{s}$) of solid API powders MET, HCT, NAC, ASA, and caffeine was calculated using the contact angle θ between the API powder and two reference liquids, ethylene glycol and α-bromonaphthalene with known surface tensions γ_l_. A Krüss Tensiometer K100 (Krüss GmbH, Hamburg, Germany) equipped with a balance was used. The cylindrical tube with an inner diameter of 12 mm was filled with the API powder, using a funnel to ensure the uniform transfer of the powder (1200–2000 mg, depending on the bulk density of the API powder) into the cylinder. The filled cylinder was then gently tapped five times to obtain a uniform powder bed. The reference liquid was filled in a cylindrical glass container. A membrane separated the interface between the API powder and the reference liquids. The measurement time was 120 s at 20 °C. The increase in weight was measured as a function of time. The Washburn equation (5) was used to calculate the contact angle θ [22]

(5) $$\frac{m^{2}}{t}=\;\frac{c*\;\rho^{2}*\;\gamma *cos\theta }{\eta }$$

where m^2^ is the squared mass gain, t is the measurement time, c is the capillary constant of solid powder, ρ is the density of the liquid, γ is the surface tension of the liquid, θ. is the contact angle, and η is the viscosity. The capillary constant c was calculated with the dispersive and low energy liquid n-hexane, which has a contact angle θ of zero [23]. The constant was used for the determination of the contact angle θ of the solid API.

The contact angle θ between ethylene glycol with its polar and dispersive part and the API powder was calculated. The contact angle θ  between α-bromonaphthalene with its dispersive part and the API powder was analyzed in the same way. The dispersive part is linked to the vdW forces. The polar part is related to the hydrogen-bonding and the dipole-dipole interactions [24]. The Owens and Wendt approach is a standard method for calculating the surface energy of a solid γ_s_ from the capillary constant c and the θ is received from the Washburn Equation (5) with two reference liquids. The model of Owens and Wendt neglects the equilibrium pressure π, i.e., the adsorbed vapor of the liquid on the solid [24].

The measured contact angles θ of α-bromonaphthalene and ethylene glycol were taken to examine the surface energy$\;\gamma_{s}$ and the polar ($\gamma_{s\;}^{p}$) and dispersive ($\gamma_{s\;}^{d}$) part of API powders. Table 1 listed the material properties of the reference liquids.

An approach to calculate the solid surface energy$\;\gamma_{s}\;$with its geometric average of polar $\gamma_{s}^{p}$ and dispersive parts $\gamma_{s}^{d}$ with two different reference liquids is described in Owens and Wendt, 1969 [24]

(6) $$\gamma_{\mathrm{sl}}=\;\gamma_{s}+\;\gamma_{l}-2*\left(\sqrt{\gamma_{l}^{d}*\;\gamma_{s}^{d}}+\;\sqrt{\gamma_{l}^{p}*\;\gamma_{s}^{p}}\right)$$

where $\gamma_{l}^{p}\gamma_{s}^{p}$ is the product of the polar parts of liquid and solid and $\gamma_{l}^{d}\gamma_{s}^{d}$ is the dispersive term. Bringing Equations (4) and (6) together, a linear equation with the intercept of $\sqrt{\gamma_{s}^{d}}$ and the slope of $\sqrt{\gamma_{s}^{p}}$ is attained:

(7) $$\frac{\gamma_{l}*\left(\cos\theta +1\right)}{2*\;\sqrt{\gamma_{l}^{d}}}\;=\;\sqrt{\gamma_{s}^{p}}*\;\frac{\sqrt{\gamma_{l}^{p}}}{\sqrt{\gamma_{l}^{d}}}+\;\sqrt{\gamma_{s}^{d}}$$

Consequently, the sum of the calculated $\gamma_{s}^{p}$ and $\gamma_{s}^{d}$ is the total surface energy $\gamma_{s}$ in mJ·m^−2^. Besides, tensiometry measurements provided information about the water sorption behavior of coated MPS. The pretreatment of the coated MPS to examine the water sorption behavior was equally as described above to calculate the surface energy, except for using 1000 mg of each sample, using purified water as test liquid, and the length of 3600 s for tensiometry measurements.

#### 2.2.6. Investigation of the Surface Tension of Liquid Coating Material via the Pendant Drop Method

A Krüss EasyDrop device (Hamburg, Germany) equipped with a halogen bulb light source, a monochrome interline CCD (25/30 fps) camera, and the DSA1 software was used. The determination of surface tension of the molten coating material $\gamma_{l}$ was performed via the pendant drop method, an image analysis based on the Young-Laplace fit, in which gravitational forces, surface tension, and interfacial tension influence the drop shape [28].

The preheated (90 °C) syringe and needle with a diameter of 1.8 mm guaranteed the controlled dosing of a single drop of the melted coating material at 90 °C. The density ρ of the melted coating material was determined in a measuring cylinder and used for the calculation of the surface tension $\gamma_{l}$.The calculation of the polar part $\gamma_{l\;}^{p}$ and dispersive part $\gamma_{l\;}^{d}$ of liquid coating needed a pre-step of measuring the contact angle θ between the melted coating and the completely dispersive solid surface Polytetrafluoroethylene (PTFE) with the surface energy 19 mJ·m^−2^ [29].

#### 2.2.7. Investigation of the Surface Energy of Solid Coating Material Using the Pendant Drop Method

The Krüss EasyDrop was used to image a goniometric sessile drop of the test liquid α-bromonapthalene (dispersive) and ethylene glycol (polar and dispersive) on a solid surface. This method calculates the contact angle θ at the interface between the drop shape of the test liquid and the defined baseline of the solid surface.

The determination of the surface energy of the solid coating material PPP and PS 65 $\gamma_{s}\;$required a pretreatment involving melting and recrystallization of the lipid on a glass plate. A syringe with a needle of 0.5 mm guaranteed the controlled dosing of each drop of the reference liquids α-bromonapthalene and ethylene glycol. The measurement of at least 10–15 drops was conducted. The surface energy $\gamma_{s}$ was determined via the Owens and Wendt method by using the measured contact angles θ between the reference liquid and the solid coating material [24].

## 3. Results and Discussion

### 3.1. In Vitro Release Profile of HCT and MET

**Figure 2:**
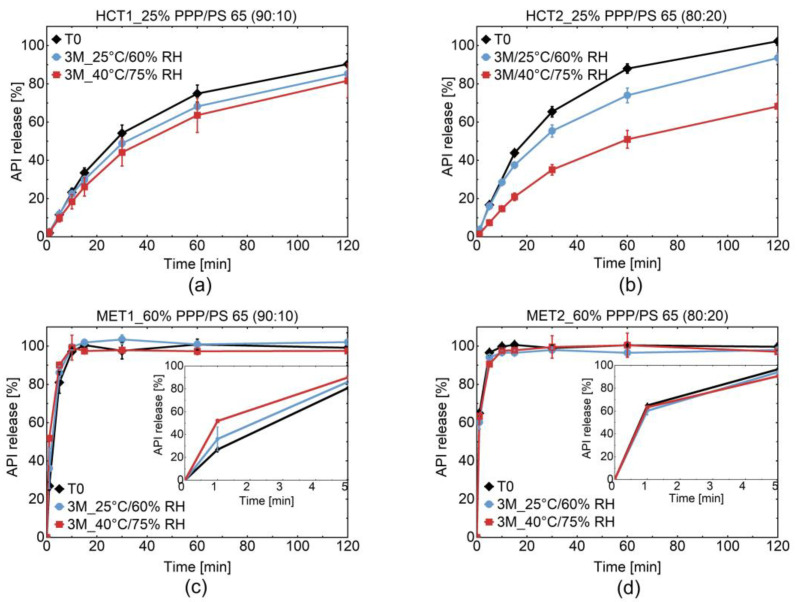
In vitro API release of coated HCT and MET MPS with PPP and 10% (**a**,**c**) and 20% (**b**,**d**) of PS 65 in the dissolution media 0.1 N HCl, respectively.

Figure 2 depicted the in vitro release alteration of coated MPS after three months of storage under long term and accelerated conditions (25 °C/60% RH and 40 °C/75% RH), respectively.

Generally, a decelerated release of HCT was observed after storage, which was more notable in particles coated with PPP and 20% of PS 65 after three months of storage under accelerated conditions. The more hydrophilic MET coated with PPP and 10% of PS 65 presented an accelerated release after storage under the same condition. The similarity factor f_2_ was calculated according to the FDA Guideline to compare the release profile of each batch after storage with the initial release profile using the following equation [30] (Table 2):

(8) $$f_{2}=50*\log\left\{{\left[1+\;\frac{1}{n}\overset{n}{\underset{t=1}{\sum }}{\left(R_{t}-T_{t}\right)}^{2}\right]}^{-0.5}*100\right\}$$

where n is the number of dissolution time points and$\;R_{t}$ and $T_{t}$ are the average percentages of dissolution rates of the two dissolution curves at the time point t. If f_2_ lies between 0 and 49, there is a significant difference between the two release profiles. According to the FDA guideline, only one additional measuring time point after 85% release should be included in the calculation [30]. The calculation of the f_2_-value of HCT1 and HCT2 was performed with all measurement points. Due to the hydrophilicity of MET, the calculation of MET1 was limited to three (1, 5, 10 min) and MET2 to two (1, 5 min) measurement points.

In Table 2, it can be observed that in all cases, the initial release profiles are similar to those after three months of storage under long term conditions, except in the case of the MET1 MPS batch (f_2_ = 41.13). During storage at 40 °C and 75% RH, a significant decrease for HCT2 MPS (f_2_ = 27.25) and a significant increase for MET1 MPS (f_2_ = 40.58) were notable. Due to the hydrophilicity of MET and higher amount of emulsifier (20% PS 65) in the coating of MET2 MPS, alteration in the API release after storage was not clearly pronounced compared to HCT MPS. The Higuchi model based on the Fickian diffusion is well-described in the literature and was identified as the best model to describe the release kinetics of HCT1 and HCT2 MPS [31,32,33,34].

The plot’s slope described the correlation between the percentage of the API release and the square root of time. The release constant can then be extracted from the plot’s slope. The release constant and the regression coefficient (R^2^) are depicted in Table 3. The change in the release constant K after storage was more expressed for HCT2 MPS with 20% of PS 65.

The duality in the alteration of API release after storage was investigated, taking into account the water solubility of the different APIs and coating materials depicted in Figure 1, the water sorption behavior of coated MPS, and the surface tension between API and coating under stress conditions.

### 3.2. Water Sorption Behavior

**Figure 3:**
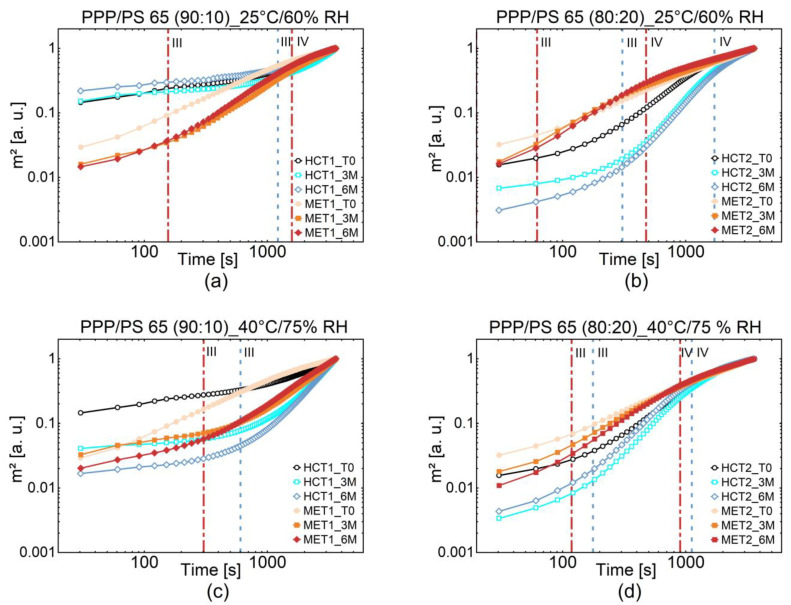
Normalized water sorption curves of coated HCT1 and MET1 with 10% of PS 65 (**a**,**c**) and HCT2 and MET2 with 20% of PS 65 (**b**,**d**) plotted in log scale at 25 °C/60% RH (**a**,**b**) and 40 °C/75% RH (**c**,**d**). The vertical lines mark the transition of the water sorption stages of six months of storage for HCT MPS (**^……….^**) and MET MPS (— - —).

The water sorption and wettability of the coated MPS with 10% (HCT1 and MET1) and 20% (HCT2 and MET2) of PS 65 were investigated right after the HMC process, after three months and six months of storage under the long term and accelerated conditions (25 °C/60% RH and 40 °C/75% RH), respectively. The change in water sorption during storage as a function of temperature and relative humidity indicates the surface changes of the coated MPS and the resulting consequences. Therefore, the capillary flow, which is influenced by water’s capillary pressure through a homogeneous powder bed separated with a membrane, was investigated. The capillary pressure depends on the porosity of packing, density, specific surface area, and wettability of powder [35]. In an ideal conception, the powder bed is an arrangement of parallel-oriented capillaries with a constant radius, in which the capillary rise of water occurs due to attractive forces between the liquid and the capillary wall [35,36,37]. The capillary water flow was measured gravimetrically as a function of time (squared weight gain per time) using the modified Washburn method, described in Section 2.2.5, where the mass m of the water is used instead of the capillary flow height h since there is a linear relationship between h and m [38,39]. The normalized water sorption curves were plotted in log scale to observe subtle alterations in the water sorption behavior (Figure 3).

A theoretical water sorption curve contains four different stages. The following equation describes every stage

(9) $$m^{2}=a+b*\;t^{c}\;\;$$

where $m^{2}$ is the normalized square weight gain, a is the y-section, b the slope, t the time of water uptake, and c a specific exponent. In the first stage, the weight gain m has a proportional dependency to the time $t^{2}$ ($m^{2}$ ~ $t^{4}$), where inertia forces of the initial liquid rise like the friction force, the entrance pressure loss, and the curvature of the free surface in the liquid dominate the water sorption [35,36]. The initial liquid height influences this regimen [23,36]. The initial liquid height in this study was 2 mm. Therefore, the first regimen could not be observed, due to the defaulted initial height at the beginning of the experiment.

The second stage is described by a linear relationship between m and t and is characterized by convective losses at the tube entrance ($m^{2}\;\sim \;t^{2}$). The hydrophilic MPS MET1 and MET2 nearly oppress this stage, and the water sorption behavior is dominated by the third stage, where the Lucas-Washburn behavior occurs. Viscous and friction forces counteract the capillary driven flow forces [36]. There is a linear increase of the weight time curve, where m is proportional to the square root of time t ($m^{2}\;\sim \;t$). The last stage is determined by the dominance of gravity forces leading to an increase in liquid mass uptake close to zero [35]. Looking at the most interesting stage III, the Lucas-Washburn behavior, MET MPS with the more hydrophilic core presents a faster water uptake than the HCT MPS with the more hydrophobic core initial value. As an indicator, stage three begins at an early stage, and the curve flattens towards the end. The curve gradually approaches equilibrium, and consequently, no liquid is further absorbed. This phenomenon is observed for MET2 MPS at the initial value. A higher proportion of PS 65, therefore, reinforces this behavior. The amount of PS 65 plays a vital role in water sorption behavior, which can be explained by its HLB value of 10.5. This effect can be seen by comparing the water uptake curves of HCT1 and HCT2 as well as MET1 and MET2 MPS at the initial value (Figure 3). Interestingly enough, stage III of water sorption is reached earlier for MET MPS stored for 6 months at 25 °C and 60% RH in comparison to MET MPS stored at 40 °C and 75% RH. The opposite phenomenon can be observed in the more lipophilic HCT MPS, where stage III begins later for coated MPS stored 6 months at 25 °C and 60% RH than at 40 °C and 75% RH.

Additionally, stage IV was not exceeded by HCT1 and MET1 MPS after storage at 40 °C/75% RH. The higher the capillary flow, the faster is the transition from stage I to IV. As described above, stage I was not observed due to the defaulted initial height at the beginning of the experiments.

The relative humidity during storage affects the water sorption of stored samples since there is an equilibrium between the water vapor attachment and the liquid wetting [40,41]. More water vapor is already attached at the surface at higher relative humidity during storage, leading to a decreased sorption rate or rather a water sorption capacity [40]. The increase of the emulsifier PS 65 may enforce the water vapor uptake during storage. Please note that the coated MPS were stored under long term and accelerated conditions in open containers.

The altered water sorption capacity of HCT and MET MPS can have an impact on their dissolution rate. The dissolution mechanism includes, thereby, three steps. First, there is a diffusion of dissolution medium through the coating towards the core, followed by dissolving the API, and consequently, the diffusion of dissolved API towards the dissolution media. In general, the second step controls the release rate except for extremely insoluble APIs [31]. In this case, the solubility of API determines the release rate. After storage at 40 °C and 75% RH, the altered water sorption capacity can affect the diffusion from the bulk medium through the coating layer to the API and vice versa. APIs with a low solubility are characterized by a lower diffusion rate according to a lower concentration gradient. The concentration gradient between the MPS interface and the surrounded medium determines the diffusion and, thereby, the release rate [31].

### 3.3. Small-Angle X-ray Scattering

Given the evidence for the stable ß polymorphic form of PPP induced by the addition of PS 65 right after the HMC process, examining other factors like crystal growth that can impact the instability of release rate seems warranted. By definition, crystal growth means the inclusion of lipid molecules on the growing surface of lipid crystals [42]. The effect of external factors on the nucleation and crystal growth of lipids is described in the literature [42,43]. Examples are environmental and processing factors such as dynamic temperature variations, shear and sonication forces, pressure, and additives as “minor” components in the lipid composition. Additives, based on their structure, can promote or prohibit either the nucleation or the crystal growth or both. Their solubility can determine the critical concentration of additives affecting the crystallization in the super-cooled lipid [42]. Considering this factor, low concentrations of below 0.1% may be sufficient for affecting the nucleation, and higher concentrations are required for affecting the crystal growth [43].

**Figure 4:**
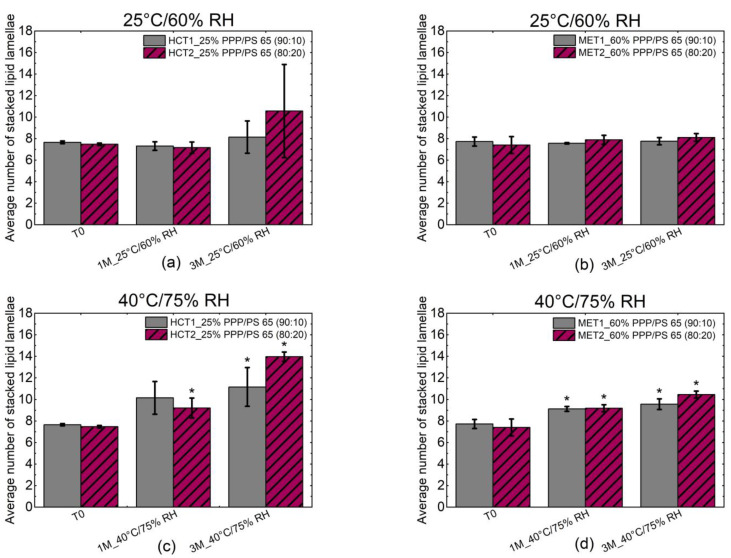
Average number of stacked lipid lamellae of PPP after storage at 25 °C/60% RH (**a**,**b**) and 40 °C/75% RH (**c**,**d**) evaluated via Scherrer Equation (2). * *p*-value (T0/1M, T0/3M or 1M/3M) < 0.05: statistically significant.

In this study, Figure 4 pointed out the temperature-induced crystal growth.

One of the influencing factors on the slowed release of HCT after storage under accelerated conditions might be the crystal growth of PPP, resulting in a more hydrophobic surface and consequently the phase separation between PPP and PS 65. Please note that PS 65 was in its liquid state during the storage because of its melting point at 33.3 °C. We previously described this phenomenon in Lopes et al., 2017 [3]. However, there are additional causes of the altered release since the more hydrophilic MET MPS showed accelerated release after storage.

The properties of core materials and their interaction with the phase separation of the binary coating during storage are described below.

### 3.4. Monitoring Phase Separation of the Coating Mixture via Confocal Raman Spectroscopy

To investigate the phase separation of the binary-lipid based system of PPP and PS 65, confocal Raman spectroscopy with horizontal and depth profile scanning was used. The calculation of PPP and PS 65 amount in the mixture implied the whole pretreated spectra range (1525–400 cm^−1^). The reference spectra PPP and PS 65 distinguished very strongly in the range between 770 and 900 cm^−1^. A peak at 890 cm^−1^ was found because of the CH_2_ rocking vibrations (890–740 cm^−1^) and was specific for both PPP and PS 65 [15]. At the lower wavenumber side of this peak, the characteristic shoulder of PS 65 appeared between 845 and 765 cm^−1^ and might correspond to the tetrahydrofuran molecule of PS 65 with its CH_2_ deformation vibrations (860–760 cm^−1^). The C-O stretching vibrations of the tetrahydrofuran also caused a strong peak at 1100–1075 cm^−1^, and O-CH_3_ stretching parts (1315–1195 cm^−1^) can be attributed to PS 65 at the peak 1245 cm^−1^ [15]. O-CH_3_ stretching parts (1315–1195 cm^−1^) can be attributed to PS 65 at the peak 1245 cm^−1^.

The peak of PPP in 1337 cm^−1^ can be explained by the ester group with CH_2_ wagging vibrations (1385–1335 cm^−1^) or CH_3_ rocking vibrations (1195–1135 cm^−1^) [15]. Preliminary trials showed that measuring the MPS could not provide reliable spectra due to a low signal-to-noise ratio. Therefore, the API powder was pressed into 18 mm tablets, which fulfilled the predefined critical resistance to crushing not less than 60 N with the Korsch single punch press (Korsch AG, Berlin, Germany). The tablet then was coated with 100 µL of liquid coating material consisting of 50% PPP and 50% PS 65 to facilitate the tracking of the two components. The coating thickness was approx. 390 µm. Within this approach, it is assumed that the surface morphology and porosity have a negligible influence on the differences between the behavior of separated PS 65 in the system. Although there was a change in the surface properties of pressed tablets compared to MPS, it was possible to track the trend of the mobility of PPP and PS 65 in the coating mixture.

**Figure 5:**
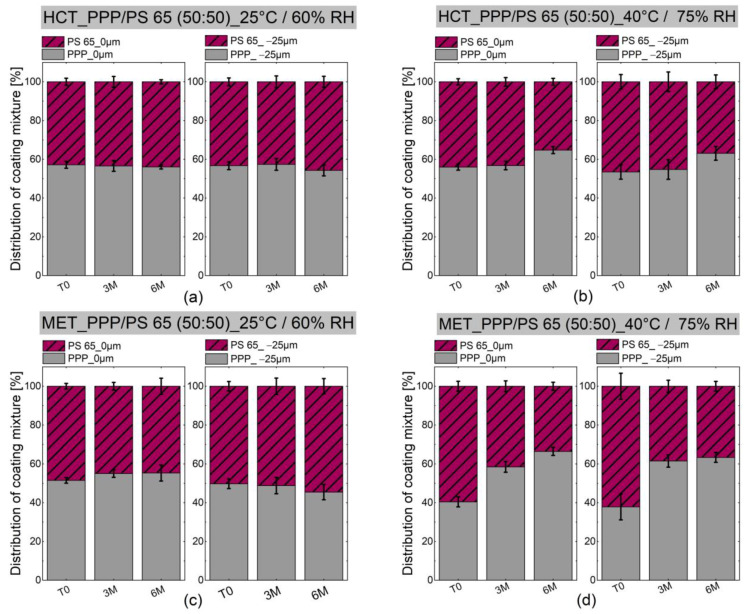
Distribution of the coating components PPP and PS 65 within the coating mixture on a pressed and coated tablet on the surface and 25 µm below the surface evaluated via confocal Raman spectroscopy: (**a**) HCT and (**c**) MET tablets stored under 25 °C/60% RH and (**b**) HCT and (**d**) MET tablets stored under 40 °C/75% RH.

As described in Section 2.2.4, a measuring grid of 3 by 3 spectra (*n* = 9) on the coating surface and a depth scan of a grid of 3 by 3 spectra 25 µm below the surface were recorded. The amount of PPP and PS 65 in the tablet coating mixture was calculated using Equation (3). The data are depicted in Figure 5.

As depicted in Figure 5, any significant phase separation of PPP/PS 65 could be observed neither for HCT nor for MET tablets stored under 25 °C/60% RH for three months and six months, respectively. There was also no significant PPP/PS 65 phase separation for HCT tablets after three and six months of storage under 40 °C/75% RH.

In contrast, MET tablets showed an increase in PPP and a decrease in PS 65 regions on the surface of the tablet and 25 µm below the surface during storage at 40 °C/75% RH, which is considered an indicator of phase separation, which can favor the release alteration. These findings are in good agreement with the accelerated water sorption of coated MET MPS and accelerated release of MET from coating after storage at 40 °C and 75% RH. The effect of phase separation leads to a polydisperse mixture of two phases and can be explained by the phenomenon of Ostwald-ripening since the mixture is not in its lowest energy state [44]. This effect occurs when many small particles merge into fewer large particles to reduce the free energy of the system and reach the thermodynamic equilibrium [44,45].

### 3.5. The Free Surface Energy of API Core and Coating Material

The surface energy of APIs was determined using the Washburn method to investigate how energetically favorable is the joint between the API core and the molten coating and thus the affinity to each other. The Washburn method is an indirect measurement technique for recording the mass uptake of a liquid in a cylinder filled with a powder [37]. Although the pendant drop method is faster than the Washburn method and less reference liquid is necessary, pre-trials showed that the latter method is the more promising method for this purpose. With the aid of the Owens-Wendt method and the investigated contact angles between the API and the reference liquids, the free surface energy of the API powder $\gamma_{s}$ was calculated. The surface energy $\gamma_{s}$ is an expression of the excessed energy on the surface and influence the wettability of solid surface [19,46]. Besides, it is necessary for the calculation of the adhesion energy $W_{\mathrm{sl}}$ in Section 3.6.

The calculated capillary constant c with the reference liquid n-hexane and the measured contact angles θ of the solid powders are summarized in Table 4.

**Figure 6:**
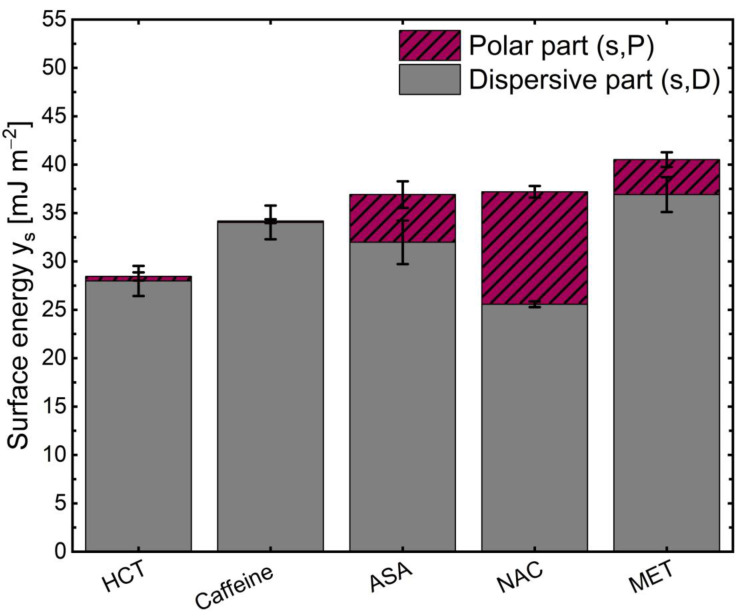
Calculation of the free surface energy of API $\gamma_{s}$ by using the Washburn method.

The total free surface energy of the different APIs $\gamma_{s}$ is depicted in Figure 6. Furthermore, the graph demonstrates the estimated polar $\gamma_{s}^{p}\;$and dispersive part $\gamma_{s}^{d}\;$of the free surface energy $\gamma_{s}$.

Figure 6 represents the fact that MET has the highest surface energy $\gamma_{s}$ and HCT the lowest. This is in good agreement with the higher polarity of MET than HCT, as can be seen from the water solubility and logP values of these two molecules, depicted in Figure 1. The higher surface energy $\gamma_{s}$ for the more hydrophilic substances could be explained by the higher proportion of polar forces. Both the secondary forces, the van der Waals forces, and the hydrogen-binding forces contribute to a surface with strong adhesive forces [24].

The surface tension of the melted coating $\gamma_{l}$ was evaluated via the pendant drop method. The densities ρ of PPP, PS 65, and two mixtures with 10 and 20% of PS 65, as well as the surface tension ($\gamma_{l}$) of the melted materials, their polar $\gamma_{l}^{p}$ and dispersive $\gamma_{l}^{d}$ parts are summarized in Table 5.

Comparing the surface tensions $\gamma_{l}$ in Table 5, the polar part of the pure emulsifier PS 65 with an HLB value of 10.5 is slightly higher than the surface tension of pure PPP. This phenomenon can be explained by the larger polar part of the molecule, as depicted from the chemical structures in Figure 1. However, considering the relatively high standard deviations, the addition of PS 65 in the mixtures does not have a distinctive impact on the surface tension of the coating.

### 3.6. Cleavage Theory

Wettability is the liquid’s aptitude to come into contact with a solid surface, where adhesive and cohesive forces are involved [20]. A proper and stable wetting behavior is given when adhesive forces merge two macroscopic bodies [20].

The formation of new surfaces of two reversible bodies in contact in equilibrium means the free energy difference per unit area, i.e., the free energy of the adhesion ΔG (mJ·m^−2^) or thus the work of adhesion $W_{\mathrm{sl}}$ (mJ·m^−2^) [20,47]. The adhesion energy $W_{\mathrm{sl}}$ between the API core s and the molten coating material l was calculated using Equation (10)

(10) $$\Delta G=W_{\mathrm{sl}}=\;\;\gamma_{l}+\;\gamma_{s}-\;\gamma_{\mathrm{sl}}$$

where $\gamma_{l}\;$is the free surface tension of molten coating material per unit area (mN·m^−1^), $\gamma_{s}$ is the free surface energy of API per unit area (mJ·m^−2^) and $\gamma_{\mathrm{sl}}\;$is the interfacial tension (mN·m^−1^). The interfacial tension $\gamma_{\mathrm{sl}}\;$was calculated with Equation (6). During storage of coated MPS under accelerated conditions, the stress factors temperature and relative humidity can affect the interfacial tension $\gamma_{\mathrm{sl}}\;$and the work of adhesion $W_{\mathrm{sl}}$ between the API core and the coating and the system tries then to minimize its interfacial tension $\gamma_{\mathrm{sl}}\;$and the overall surface energy $\gamma_{s}$. If the formation of a new interface with the stress factor is energetically preferred, the cleavage of the API/coating bond could be the result. The condition of 40 °C/75% RH was considered as a stress factor, and the work ($W_{\mathrm{lws}}$ (mJ·m^−2^)) to cleave the coating from API in this condition and thus the affinity between the API core and the coating material was calculated by the following equation:

(11) $$W_{\mathrm{lws}}=\;\;W_{\mathrm{sl}}+\;W_{\mathrm{ww}}-\;W_{\mathrm{lw}}-\;W_{\mathrm{sw}}\;\;$$

This approach is based on the comparison of the work, which would be required to break all the existing interfaces ($W_{\mathrm{sl}}$ + $W_{\mathrm{ww}}$) and the work to create new surfaces between the single MPS components API and coating and the stress factor water vapor ($W_{\mathrm{lw}}$ + $W_{\mathrm{sw}}$). The required work of adhesion $W_{\mathrm{sl}}$ in mJ·m^−2^ to break the existing bond between the API and the coating is calculated with Equation (10). The necessary work to overcome the intermolecular forces within the stress factor to create new formations with the single MPS components is equal to the work of cohesion of water vapor droplets ($W_{\mathrm{ww}}$ (mN·m^−1^)) [20,47]:

(12) $$W_{\mathrm{ll}}=\;\;2*\gamma_{l}$$

According to Pérez-Díaz et al. 2012 [48], the surface energy of water molecules decreased with a decrease in relative humidity. Pérez-Díaz et al., 2012 investigated in their study surface energy of approximately 70 mN·m^−1^ applied for 40 °C/75% RH. The cohesion energy of the water vapor $W_{\mathrm{ww}}$ corresponds to twice the surface energy of it, namely 140 mN·m^−1^. This value was used for the following calculations. Finally, the work of the creation of new interfaces between the water vapor and the lipid coating ($W_{\mathrm{lw}}$ (mN·m^−1^)) and the water vapor and the API ($W_{\mathrm{sw}}$ (mJ·m^−2^)) was calculated with Equation (10).

**Figure 7:**
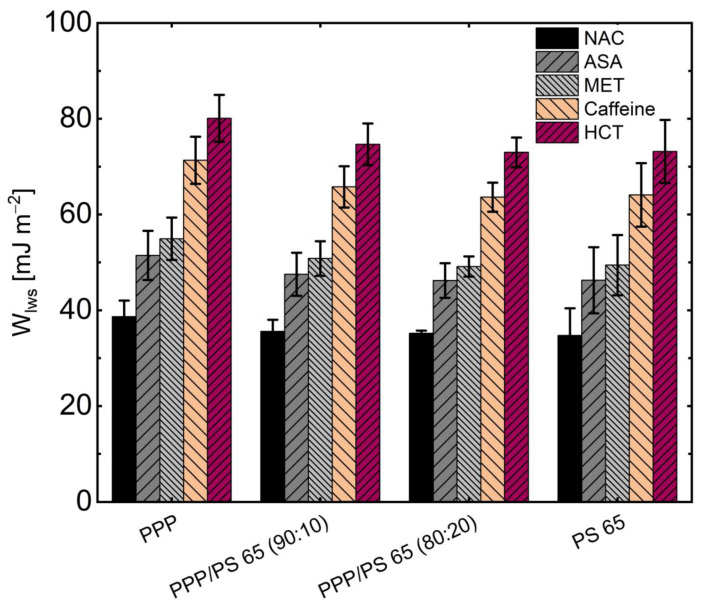
Work $W_{\mathrm{lws}}$ to cleave coating from the API in the presence of the stress factor.

The results are depicted in Figure 7. The graph shows that higher work is required to affect the existing interface between the coating and the more lipophilic API such as HCT and caffeine in the presence of the stress factor. Two lipophilic surfaces (lipophilic API and coating material) have more vital forces of attraction to each other in the presence of relative humidity. NAC, ASA, and MET had lower adhesion work with their hydrophilic behavior and appeared to have lower resistance to the stress factor. In such a case, attractive forces can then convert to more repulsive forces, facilitating the phase separation [20]. When comparing NAC and MET, it is noticeable that the cleavage work seems to decrease as the polar part of the surface energy increases (Figure 6). There was no significant difference in the surface tensions of the coating compositions and the pure coating material PPP and PS 65 (Table 5) and also no difference in their cleavage work (Figure 7).

As mentioned previously, in the stress condition, 40 °C/75% RH, the emulsifier PS 65 is in a molten state because of its low melting temperature of 33.3 °C, whereas PPP is solid. As expected, the surface energies of the PPP and PS 65 in their solid form at room temperature (39.05 ± 0.78 and 40.62 ± 1.08 mJ·m^−2^, respectively) were higher than those of molten PPP (23.84 ± 2.37 mN·m^−1^) and molten PS 65 (26.41 ± 2.55 mN·m^−1^) at 90 °C, which can be explained, for instance, by the weakening of the van der Waals interaction forces [20,49]. The interfacial tension of PPP and PS 65 both in a molten state ($\gamma_{\mathrm{ll}}$) was 0.31 mJ·m^−2^ and the interfacial tension between the solid PPP and molten PS 65 ($\gamma_{\mathrm{sl}}$) (correlated to the state of coating stored at 40 °C/75% RH) was 3.35 mJ·m^−2^. The latter state has a significantly higher interfacial tension, which, combined with crystal growth of PPP, can favor the phase separation of PPP and PS 65 during storage. The phase separation is based on the Ostwald ripening effect, where a thermodynamic equilibrium is achieved by reducing the interfacial area [44].

The phase separation of the binary lipid-based system of PPP and PS 65 and the effect of temperature has been described in Lopes et al., 2017 [3]. Please note that the core material NAC was used in that work and was hot melt coated with the binary mixture of PPP and PS 65. The consequence of phase separation was the faster release of NAC from coating after storage under accelerated conditions. This behavior agrees with the findings in the current study, where lower adhesion work is calculated for hydrophilic NAC, ASA, and MET as core materials. The faster release of MET from coating after storage under accelerated conditions as described above (Figure 2c).

Our unpublished data also showed the faster release of ASA from the same coating after storage under accelerated conditions. On the contrary, the affinity between the lipophilic core with lipophilic coating, combined with the crystal growth of PPP after storage at 40 °C and 75% RH, result in the deceleration of HCT release after storage (Figure 2a,b).

## 4. Conclusions

This work aimed to provide in-depth knowledge about the physical properties of core materials and their interaction with a two-phase lipid-based coating composition.

The alteration in the release profiles of core materials with different lipophilicity, coated with the same composition of PPP and PS 65, were investigated as the response to storage at 40 °C/75% RH in open containers. The release of polar molecules such as MET was accelerated, and lipophilic molecules such as HCT were decelerated after storage.

During storage at 40 °C and 75% RH, temperature-dependent crystal growth of PPP in the coating was more favored by hydrophobic HCT core. The phase separation of PPP and PS 65 was assumed due to the higher interfacial tension between the coating components during storage, combined with the crystal growth of PPP. Increased water sorption due to water vapor accumulation onto the surface was more dominant for coated polar MET particles. The cleavage work necessary to overcome the adhesion of coating composition on the core surface was calculated for each API. The accelerated release of the polar core after storage at 40 °C/75% RH was correlated with a low cleavage work and a distinctive phase separation.

In contrast, a decelerated release of the hydrophobic core was favored by the pronounced crystal growth of the lipid-based coating. APIs with different lipophilicity differ in their affinity to the coating and their distinctive phase separation. Phase separation and weak affinity seem to overrule the effect of crystal growth, resulting in a faster release in the case of more hydrophilic APIs.

## Figures and Tables

**Table 1:** Material properties of the reference liquids.

Reference Liquid	Surface Tension $\gamma_{l}$ [mN·m^−1^]	Dispersive Part $\gamma_{s}^{d}$ [mN·m^−1^]	Polar Part $\gamma_{s}^{p}$ [mN·m^−1^]	Density ρ [kg·m^−3^]	Viscosity η [mPa·s]
Ethylene glycol ^3^	48.0	29.0	19.0	1113	15.4
α-Bromonaphthalene ^1,4^	44.6	44.6	0.0	1478	4.52
n-hexane ^2,3^	18.4	18.4	0.0	659	0.31

^1^ [25]. ^2^ [26]. ^3^ [17]. ^4^ [27].

**Table 2:** Similarity factor f_2_ of release profiles (f_2_ ∈ [50, 100]: similar profiles), one-factor analysis of variance (*p*-value (T0/1 min) < 0.05: difference statistically significant).

MPS	*f*_2_ T0/3M_25 °C/60% RH	*p*-Value (1 min) T0/3M_25 °C/60% RH	*f*_2_ T0/3M_40 °C/75% RH	*p*-Value (1 min) T0/3M_40 °C/75% RH
HCT1_25% PPP/PS 65 (90:10)	87.79		59.55	
HCT2_25% PPP/PS 65 (80:20)	51.88		27.25	
MET1_60% PPP/PS 65 (90:10)	41.13	0.21	40.58	7.41 × 10^−5^
MET2_60% PPP/PS 65 (80:20)	70.71	0.10	67.54	0.13

**Table 3:** Release constant K calculated from the Higuchi model and regression coefficient (R^2^) of HCT1 and HCT2 MPS at time zero and after three months of storage at 25 °C/60% RH and 40 °C/75% RH [31,32,33,34].

MPS	$K\;[min^{-\frac{1}{2}}]$ T0	R^2^	$K\;[min^{-\frac{1}{2}}]$ 3M_25 °C/60% RH	R^2^	$K\;[min^{-\frac{1}{2}}]$ 3M_40 °C/75% RH	R^2^
HCT1_25% PPP/PS 65 (90:10)	9.96 ± 0.49	0.9951	10.31 ± 0.55	0.9943	9.12 ± 0.32	0.9969
HCT2_25% PPP/PS 65 (80:20)	12.85 ± 1.11	0.9852	10.47 ± 0.83	0.9875	7.95 ± 0.28	0.9975

**Table 4:** The calculated capillary constant c and contact angles θ of different APIs using the Washburn method.

API Powder	c (API/n-Hexane)	θ (API/α-Bromonaphthalene) [°]	θ (API/Ethylene Glycol) [°]
MET	3.2 × 10^−5^ ± 5.1 × 10^−6^	34.8 ± 4.6	45.0 ± 2.3
NAC	2.8 × 10^−5^ ± 2.7 × 10^−6^	59.0 ± 0.6	41.1 ± 1.6
ASA	3.9 × 10^−5^ ± 4.0 × 10^−6^	46.0 ± 4.8	48.0 ± 0.9
Caffeine	5.0 × 10^−5^ ± 2.9 × 10^−6^	41.6 ± 3.9	73.1 ± 2.9
HCT	2.7 × 10^−5^ ± 1.8 × 10^−6^	54.2 ± 3.0	72.5 ± 1.4

**Table 5:** Characterization of the coating material. Surface tension was calculated via the pendant drop method.

Coating Material	Density ρ [kg·m^−3^]	Surface Tension $\gamma_{l}$ [mN·m^−1^]	Dispersive Part $\gamma_{l}^{d}$ [mN·m^−1^]	Polar Part $\gamma_{l}^{p}$ [mN·m^−1^]
PPP	8467 ± 0.01	23.84 ± 2.37	21.19 ± 2.36	2.65 ± 0.80
PS 65	9350 ± 0.04	26.41 ± 2.55	21.65 ± 2.56	4.76 ± 1.89
PPP/PS 65 (90:10)	8545 ± 0.00	26.84 ± 3.44	22.54 ± 0.98	4.30 ± 0.34
PPP/PS 65 (80:20)	8654 ± 0.07	24.76 ± 1.90	19.57 ± 1.04	5.19 ± 0.13

## Data Availability

Data is contained within the article or Supplementary Material.

## Supplementary Materials

The following are available online at https://www.mdpi.com/1999-4923/13/3/366/s1, Figure S1: UV absorption spectra of HCT (a) and MET (b). Figure S2: SAXS pattern of coated MPS HCT1, HCT2, MET1, MET2 at time zero (T0), and after the storage of one month and three months at 25 °C/60% RH (a and b) and 40 °C/75%°RH (c and d). The Full Width at Half Maximum (FWHM) of the SAXS peak (001 plane) for the Scherrer equation (2) was calculated with a Gaussian amplitude function. Figure S3: Raman spectra of PPP ($I_{\mathrm{PPP}}$) and PS 65 $(I_{\mathrm{PS}\;65}$) normalized to their intensity value at (a) 903 cm^−1^ to suppress the intensity of HCT crystals and (b) 738 cm^−1^ to suppress the intensity of MET crystals, (c) Raman spectra of HCT tablet (HCT_PPP/PS 65 (50:50)) and MET tablet (MET_PPP/PS 65 (50:50)) at time zero used in Equation (3).
