# Supplementary material for: Impact of Surface Properties of Core Material on the Stability of Hot Melt-Coated Multiparticulate Systems
Source: Pharmaceutics. 2021 Mar 10;13(3):366. doi: 10.3390/pharmaceutics13030366 (PMC8001618; doi:10.3390/pharmaceutics13030366)
Supplement: Supplementary file 1 [file pharmaceutics-13-00366-s001.pdf]

pharmaceutics

Supplementary Materials: Impact of Surface Properties of Core Material on the Stability of Hot Melt Coated Multiparticulate Systems

Sonja Schertel, Sharareh Salar-Behzadi, and Andreas Zimmer

2.2.2. In vitro release profile of HCT and MET

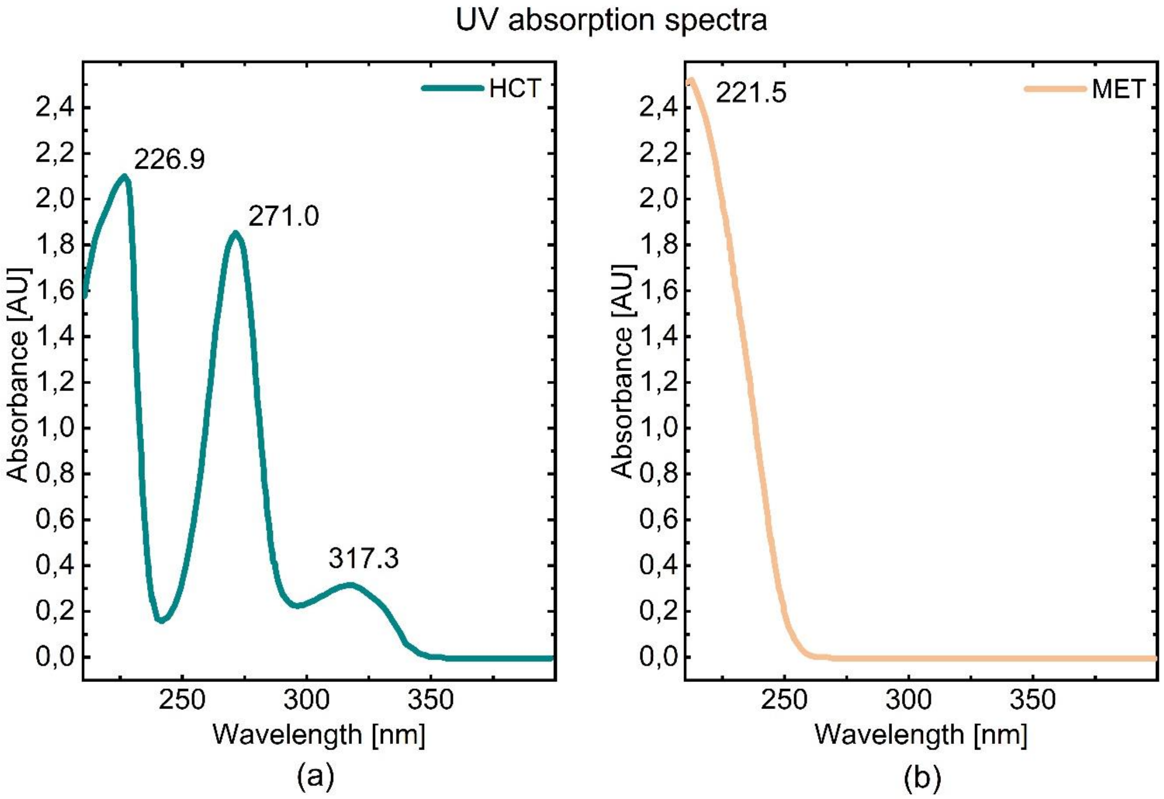

Figure S1. UV absorption spectra of (a) HCT and (b) MET.

2.2.3. Small-angle x-ray scattering

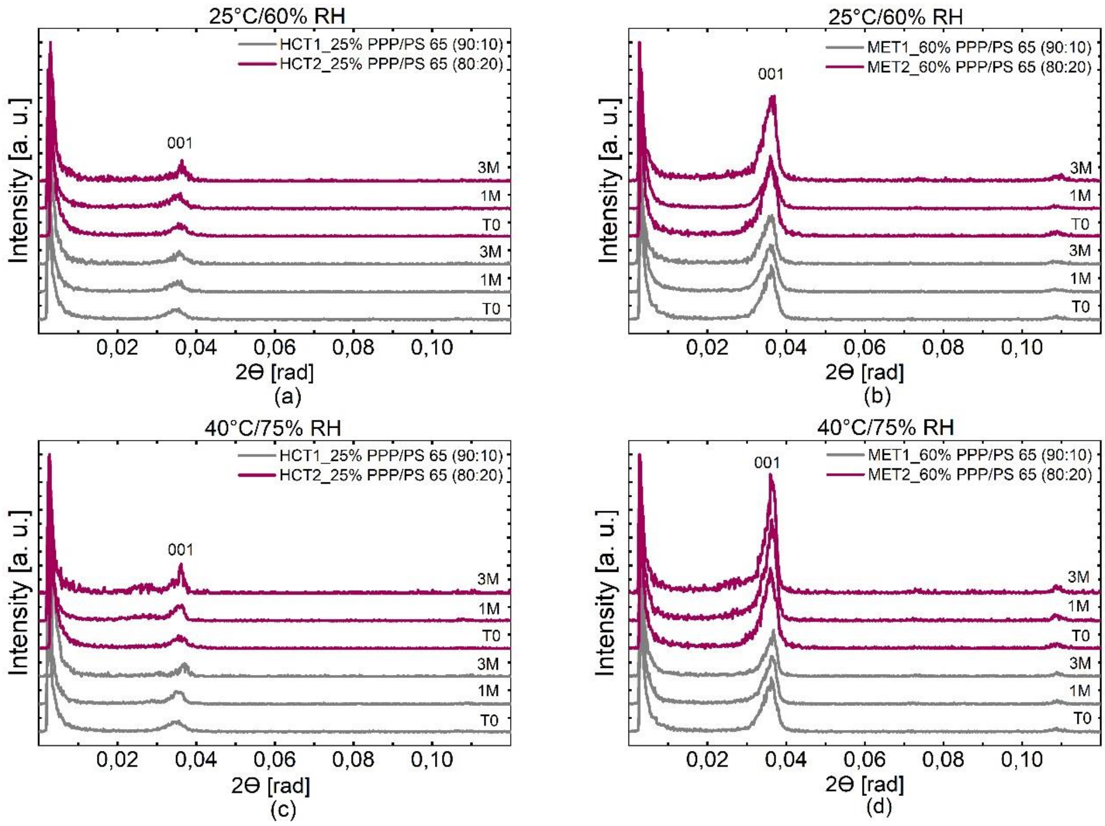

Figure S2. SAXS pattern of coated MPS HCT1, HCT2, MET1, MET2 at time zero (T0) and after the storage of one month and three months at 25°C/60% RH (a and b) and 40°C/75%°RH (c and d). The Full Width at Half Maximum ($FWHM$) of the SAXS peak (001 plane) for the Scherrer equation (2) was calculated with a Gaussian amplitude function.

2.2.4. Monitoring phase separation of the coating mixture via confocal Raman spectroscopy

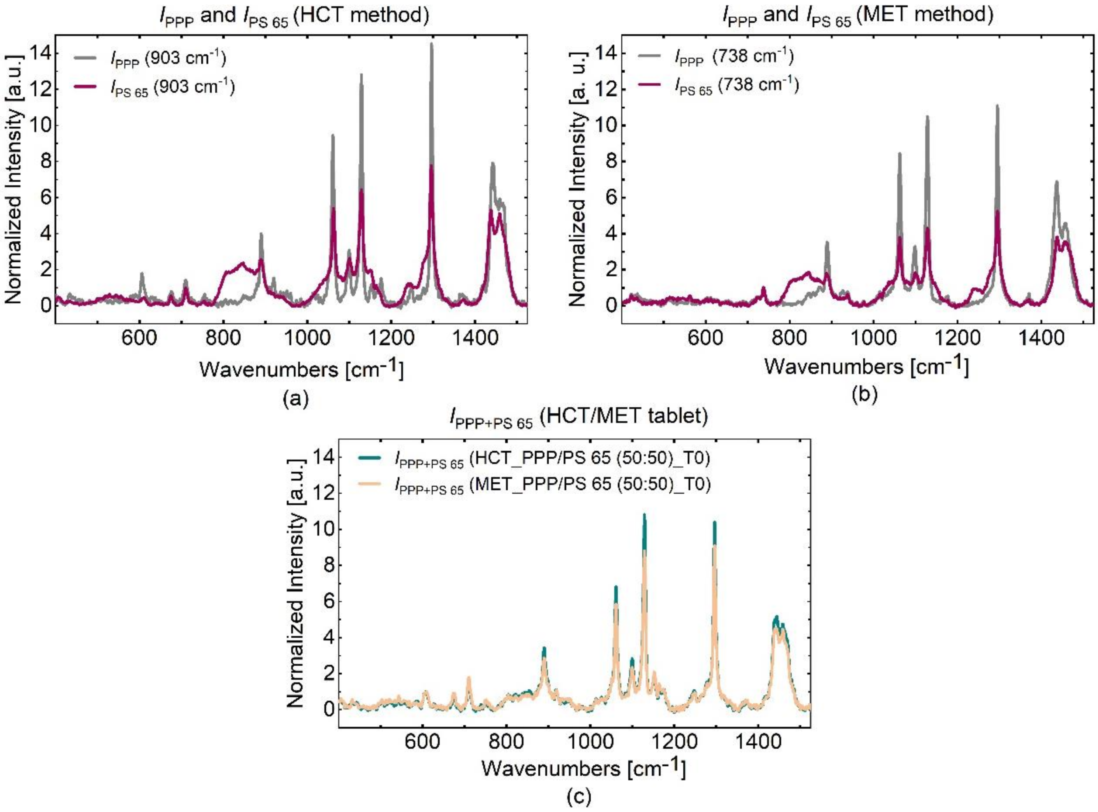

Figure S3. Raman spectra of PPP (I_{PPP}) and PS 65 ($I_{PS\ 65}$) normalized to their intensity value at a) 903 cm^{-1} to suppress the intensity of HCT crystals and b) 738 cm^{-1} to suppress the intensity of MET crystals, c) Raman spectra of HCT tablet (HCT_PPP/PS 65 (50:50)) and MET tablet (MET_PPP/PS 65 (50:50)) at time zero used in equation (3).
